# Unveiling the Phenotypic Variability of Macrophages: Insights from Donor Diversity and Pooling Strategies

**DOI:** 10.3390/ijms26031272

**Published:** 2025-01-31

**Authors:** Bartłomiej Taciak, Agnieszka Grochowska, Małgorzata Górczak, Emilia Górka, Marcin Skorzynski, Maciej Białasek, Tomasz P. Rygiel, Magdalena Król

**Affiliations:** 1 Center of Cellular Immunotherapies, Warsaw University of Life Sciences, 02-786 Warsaw, Poland; bartlomiej_taciak1@sggw.edu.pl (B.T.); agnieszkagrochowska99@gmail.com (A.G.); malgorzata_gorczak@sggw.edu.pl (M.G.); gorkaemila@gmail.com (E.G.); m.bialasek@cellis.eu (M.B.); 2 Department of Immunology, Mossakowski Medical Research Institute, Polish Academy of Sciences, 02-106 Warsaw, Poland; mskorzynski@imdik.pan.pl (M.S.); trygiel@imdik.pan.pl (T.P.R.)

**Keywords:** macrophages, BMDM, donor diversity

## Abstract

Macrophages are key players in inflammation and immune responses due to their phenotypic plasticity. This study examined the effects of pooling donor-derived macrophages on their phenotype and function, focusing on murine bone marrow-derived macrophages (BMDMs) and human monocyte-derived macrophages (hMDMs). Murine BMDMs were generated using L929-conditioned media and compared across single and pooled donors (two-to-five mice). Similarly, hMDMs cultured with M-CSF from individual donors were compared to pooled cultures. Pooling macrophages did not alter core phenotypic markers (CD11b, F4/80, CD64) or functional outputs such as cytokine secretion and nitric oxide production. In hMDMs, pooling reduced variability and led to slightly elevated or more-uniform marker expression. These findings demonstrate that pooling macrophages minimizes inter-individual variability without compromising cellular stability or function, enhancing reproducibility in immunological research while maintaining the option of single-donor studies for personalized analyses.

## 1. Introduction

Macrophages, present in all tissues, are key players in inflammation and many physiological and pathological conditions due to their remarkable phenotypic plasticity [1]. Their ability to polarize and differentiate into subtypes such as osteoclasts, Kupffer cells, or dendritic cells reflects their functional diversity in tissue homeostasis and immune responses [2]. An important perspective is the modulation of macrophage activation in specific pathological conditions to maintain cellular homeostasis. In disorders where cell proliferation is impaired, M1 macrophages can drive excessive inflammation with potentially fatal outcomes, whereas M2 macrophage-driven inflammatory disorders can lead to chronic effects and enhanced blood vessel formation [3]. Interest in macrophage-based therapies for liver cirrhosis, osteoporosis, and cancer continues to grow, emphasizing their therapeutic potential [4,5,6,7,8,9]. The importance of macrophages in laboratory testing and therapy is undeniable. Established cell lines, such as RAW 264.7, are widely used for in vitro and in vivo experiments because they provide a reliable and unlimited cell source. However, it is increasingly important to verify the characteristics of these cells between laboratories and across passages. In our previous study, we tracked the RAW 264.7 phenotype and functions from passage no. 5 to passage no. 50 and observed stability up to passage no. 30 but recommended avoiding usage beyond that to maintain reliable data [10]. 

Bone marrow-derived macrophages (BMDMs) are also commonly employed to study macrophage biology because of their ready accessibility and the ability to produce large quantities of cells. Differentiation usually requires macrophage colony-stimulating factor (M-CSF), either via direct supplementation of recombinant M-CSF or L929 cell-conditioned medium [11,12]. While recombinant M-CSF is specific, its cost and handling demands often make L929-conditioned medium the preferred method [13,14]. While it is generally accepted that L929-conditioned media can produce functional macrophages, the detailed phenotypes of these cells, compared to those generated using recombinant M-CSF, remain underexplored. Understanding the nuances of macrophage phenotypes based on the differentiation method is crucial, as they can influence experimental outcomes and interpretations. The available scientific literature describes the phenotypes of such macrophages only minimally, often relying on the expression of just two-to-three surface receptors [14,15,16,17].

Two primary sources of human macrophages are typically used in research and clinical settings: macrophages derived from peripheral blood monocytes (hMDMs) in the presence of recombinant M-CSF, and macrophages generated from human-induced pluripotent stem cells (hIPSCs) [18,19,20]. hMDMs are straightforward to produce, while hIPSC-derived macrophages require more-complex culture conditions but offer a potentially unlimited supply. To increase hMDM yields, monocytes can be collected from large-volume leukopaks, generating billions of cells [21]. For preclinical studies, monocytes are frequently obtained from buffy coats from single donors, which can provide up to 100 million macrophages [22]. A key question is whether it is appropriate to pool cells from multiple donors for a single experiment or maintain donor-specific lines, since combining cells might introduce variability. 

In this work, we provide a detailed characterization of murine and human macrophages, each cultured under specific conditions: mouse cells using L929-conditioned medium and human cells with M-CSF. We compare single-mouse versus pooled-mouse (two, three, four, or five donors) BMDMs and single-donor versus three-donor pooled human macrophages to assess how donor diversity affects phenotype, function, and yield. By exploring these different configurations, we aim to shed light on how genetic background, environmental factors, and physiological conditions shape macrophage heterogeneity. Such insights are critical for refining experimental models: pooling multiple donors can potentially minimize variability, enhance reproducibility, and strengthen the translational relevance of in vitro macrophage studies. Furthermore, we evaluate the phenotypic effects of deriving human macrophages from donors without HLA matching, thereby advancing our understanding of how genetic and immunological differences influence macrophage biology. Ultimately, this work provides valuable guidelines for designing more-robust and -informative macrophage research protocols.

## 2. Results

### 2.1. Number of Cells, Viability, and Size

**Figure 1:**
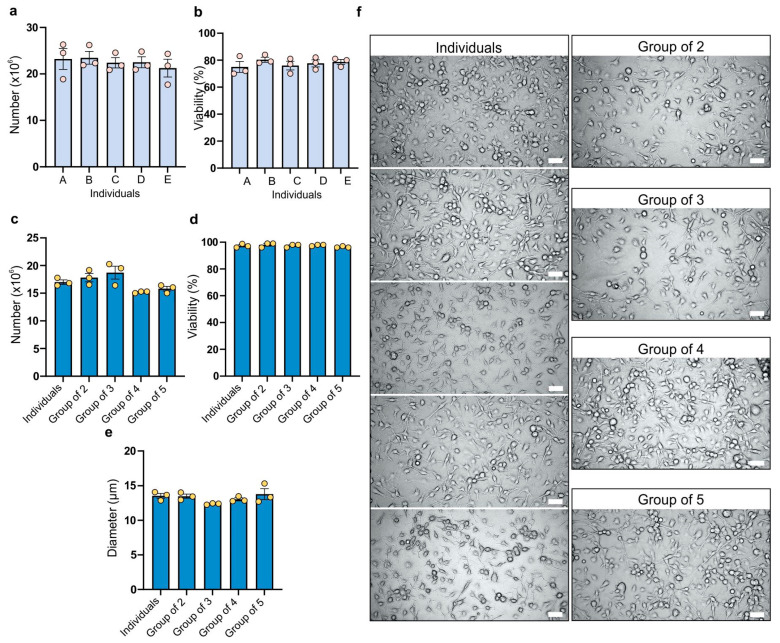
Bone marrow (**a**,**b**) and bone marrow-derived macrophage (**c**–**e**) cell characteristics: (**a**) Number of cells isolated from the bone marrow of individual mice, expressed as the mean ± SEM from three biological replicates; (**b**) Viability of cells isolated from the bone marrow of individual mice, expressed as the mean ± SEM from three biological replicates; (**c**) Number of BMDMs, expressed as the mean number of cells from individually cultured subjects and mean ± SEM cell count in various experimental combinations; (**d**) Viability of BMDMs, expressed as the mean ± SEM viability of cells from individually cultured subjects and mean ± SEM viability of cells in various experimental combinations; (**e**) Size of BMDMs, expressed as the mean ± SEM size of cells derived from individual subjects and the mean size of cells in various experimental combinations; (**f**) Representative microscopy images of BMDMs. The left panel shows BMDMs cultured individually from different subjects, while the right panel illustrates BMDMs in various experimental setups. Each image includes a scale bar representing 50 µm for size comparison. Individual data points are shown as dots. Statistical analysis was performed using one-way ANOVA with Tukey’s multiple comparisons test.

The analysis of the bone marrow cell count harvested from the femurs and tibias of BALB/c mouse females revealed that in the majority of cases the number of cells exceeded 20 million, ensuring an adequate number of cells for further experiments and consistently high numerical values (Figure 1a). Additionally, all individuals and replicates showed a viability of 70% or higher, facilitating a suitable cell culture (Figure 1b). Differences between various groups in both cell count and viability of isolated cells were not statistically significant. For cell count, the coefficient of variation ranged from 8.58% to 16.74%, while for viability it ranged from 4% to 9.3%.

The number of BMDMs obtained for each specific combination on the seventh day of culture in each case was 15 million or higher (Figure 1c) from 2 million bone marrow cells. The group of three (bone marrow cells from three mice mixed together) exhibited the highest cell count, while the group of four showed the lowest, but the differences are not statistically significant. The coefficient of variation ranged from 1.14% (group of four) to 10.87% (group of three). In all experimental combinations, a viability of 95% or higher was achieved, indicating suitability for further experimentation (Figure 1d). All tested groups showed a low coefficient of variation (ranging from 0.59% to 1.77%). BMDM size was the next factor compared between the groups (Figure 1e). The smallest size was observed in cells from the group of three, while the group with the largest size was the group of five (comprising cells from five individuals). A reduction in size compared to individually cultured subjects was evident in the groups of three and four. However, the difference in size of BMDMs was not statistically significant. The coefficient of variation ranged from 0.84% to 9.91%. The group of five showed the highest variability, while the group of three showed the lowest; however, overall those differences were small.

### 2.2. Expression of Cell Surface Markers

**Figure 2:**
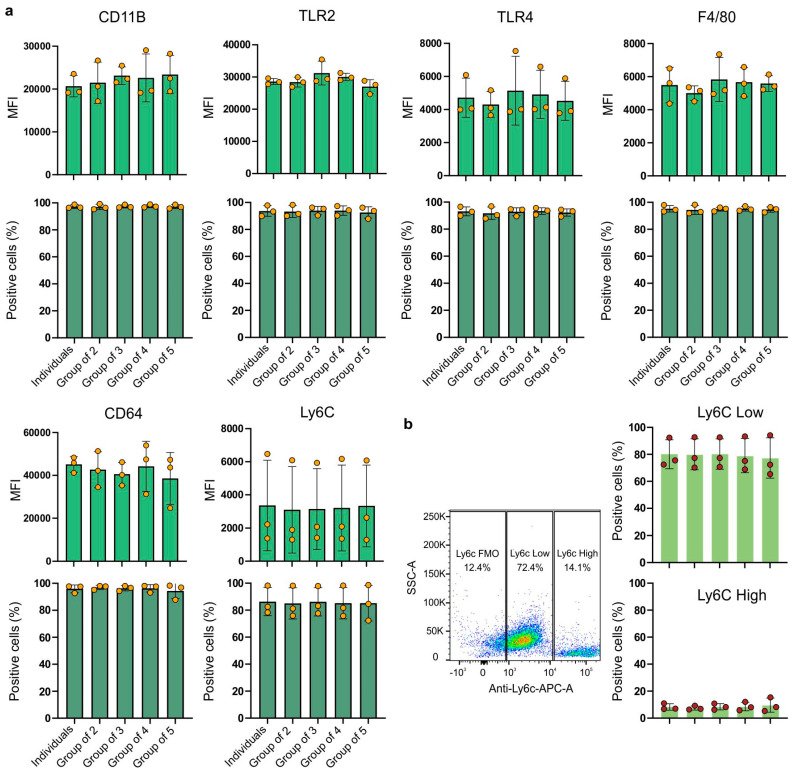
Expression of surface markers on BMDMs in experimental combinations. (**a**) The bar graphs show the mean ± SEM fluorescence intensity (MFI) and the mean ± SD percentage of positive cells for various surface markers across different groups and individuals. Each marker is represented by two graphs: the upper row for the MFI and the lower row for the percentage of positive cells. The markers analyzed include CD11B, TLR2, TLR4, F4/80, CD64, and Ly6C. (**b**) Representative scatter plot of analyzed cells showing SSC-A vs. Anti-Ly6C-APC-A. The plot displays three distinct populations based on Ly6C receptor expression: Ly6C-negative (Ly6C FMO), Ly6C-low, and Ly6C-high. The accompanying graphs on the right illustrate the percentage of positive cells for the Ly6C-low and Ly6C-high populations. Data are presented as the mean ± standard deviation (SD) from three biological replicates. Individual data points are shown as dots. Statistical analysis was performed using one-way ANOVA with Tukey’s multiple comparisons test.

High expression of the CD11b marker was observed in all experimental combinations (Figure 2a). It can be observed that the expression of the CD11b receptor (the MFI value) was slightly higher in the experimental groups compared to the cells from the group of individuals. 

The group of cells derived from four individuals showed the highest variability (24.73%). The coefficient of variation ranged from 8.9% (group of three) to 24.73% (group of four). Almost all cells across all groups expressed the CD11b receptor with coefficients of variation up to 2%. For TLR2 receptor expression, the highest MFI value was observed for the group of three (Figure 2a). The highest variability was represented by the group of macrophages derived from three individuals (group of three), with a coefficient of variation of 11.86%. The least variable was the group of individuals (with a coefficient of variation of 3.23%). Similarly to the CD11b receptor, the TLR2 receptor was also expressed by almost all cells, with negligible differences between groups. TLR4 receptor expression (Figure 2a) showed greater variation between biological replicates than between experimental groups. The highest mean fluorescence intensity (MFI) value for TLR4 expression in each group consistently originated from the same biological replicate. Despite significant differences between biological replicates, the variations between pooled and individually cultured macrophages were not statistically significant. However, they showed a large coefficient of variation, ranging from 18.05% (group of two) to 40.45% (group of three). Although there were some differences in the MFI values, nearly 100% of the cells expressed the TLR4 receptor, with a low coefficient of variation: between 2.64% and 5.06%. For the Ly6C receptor (Figure 2a), similarly to TLR4 or CD11B, it is clear that there are significant differences between biological replicates, but the differences between experimental groups remain minimal. These data suggest that while the expression levels of Ly6C may vary between donors, mixing bone marrow cells from different mice to generate macrophages does not affect the expression level. The expression of Ly6C is also characterized by a large coefficient of variation, ranging from 74.15% (group of five) to 84.17% (group of two). Additionally, there are some observable variations in the percentage of cells expressing this receptor, with a coefficient of variation ranging from 12.14% (individuals) to 15.45% (group of five).

Lastly, a new approach for assessing macrophage populations has emerged, moving beyond the traditional classification of macrophages into classically activated (M1- or LPS/IFN-γ-activated) or alternatively activated (M2- or IL-4/IL-13-activated) categories. This new understanding focuses on the expression of the Ly6C receptor, distinguishing macrophages into two main populations: Ly6C-low and Ly6C-high. This classification creates a continuum of macrophage states based on the proportion of cells expressing low or high levels of Ly6C, reflecting a more-accurate biological and in vivo state of macrophages compared to the extreme in vitro M1/M2 polarization [23]. In our study, we examined macrophage subpopulations based on their Ly6C receptor expression (Figure 2b). The mean percentage of Ly6C-low macrophages ranged from 77.3% (group of five) to 80.2% (group of three), with no statistically significant difference between groups. Similarly, the mean percentage of Ly6C-high macrophages ranged from 7.4% (group of two) to 9.6% (group of five), also showing no statistically significant difference between groups. These findings highlight the stable distribution of Ly6C-low and Ly6C-high macrophage populations in the conditions studied. 

Expression of the F4/80 receptor (Figure 2a), a characteristic receptor used as a marker for bone marrow-derived murine macrophages, was similar between the groups. The coefficients were similar for the five examined groups and ranged from 8.85% (group of two) to 22.72% (group of three). The proportion of cells expressing the F4/80 receptor was close to 100%, with a low variability and coefficients of variation ranging from 1.50% (group of three) to 3.58% (group of two). The expression levels of the CD64 receptor (Figure 2a) did not significantly vary among the different experimental groups. The experimental groups showed different coefficients of variation. The lowest variability was observed in BMDMs from the group of individuals (8.03%), while the highest variability was seen in the group of five individuals (31.39%). Again, almost all cells expressed the CD64 receptor with very low coefficients of variation, ranging from 1.58% (group of two) to 6.04% (group of five). 

The ANOVA test showed that none of the examined groups showed statistical significance. The results obtained were not statistically significant.

### 2.3. Gene Expression Analysis

**Figure 3:**
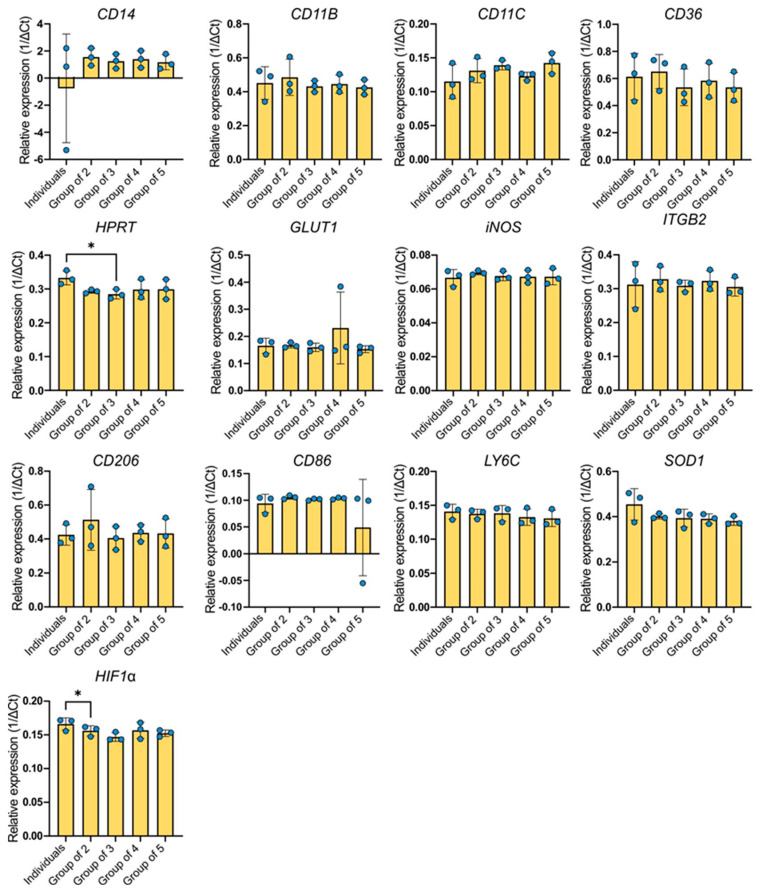
Average ± SD of relative gene expression in BMDMs under various experimental conditions. Data are presented from three biological replicates. Individual data points are shown as dots. Statistical analysis was performed using one-way ANOVA with Tukey’s multiple comparisons test; * indicates *p* < 0.05.

The gene CD14 showed the highest expression among the genes examined (Figure 3). The greatest variability in the expression level of this gene was observed in the BMDMs group derived from different individuals but cultured separately. The expression level was practically the same as that of the reference gene, for which the relative expression value is equal to one. In subsequent groups, an increase in the average gene expression was observed compared to individual expression. Except for the individuals group, the coefficient of variation was similar across all experimental groups (41.63–47.89%). However, in the single-cell group it was significantly higher at 530.3%. The ANOVA test showed no statistical significance between the combinations. 

The other genes with high expression levels were CD36, CD206, SOD1, and CD11b. Regarding the CD36 gene, the average gene expression levels in the groups were lower compared to the individuals group, with the exception of the group of two. The coefficients of variance slightly differed between the groups, ranging from 19.22% for the group of two to 27.97% for single-cultured BMDMs (individuals). An increased expression of CD206 was observed in the group of two, which also had the highest coefficient of variation (34.79%). The remaining groups were around the expression level expressed by single-cultured macrophages. The coefficients of variation ranged from 14.07% to 19.68%. Similarly, the CD11b gene was expressed in all examined groups. However, both the individuals group (21.41%) and the group of two (22.02%) exhibited the highest coefficients of variation. The other groups had approximately two-times-lower variability (7.82–10.34%). In the case of Itgb2 gene expression, the most variable group was the group of individuals (21.68%). The groups of three and four showed similar levels of expression, but the mean expression of the gene was higher in the groups of two and four. These groups had low coefficients of variation (5.39–11.04%). The expression of the SOD1 gene oscillated around a similar level as the CD206 gene. The highest expression was characteristic of individual BMDMs. The other examined groups expressed the gene at a lower level, which was very similar in each group. The individuals group exhibited the highest variability (15.35%), while the coefficients of the other groups ranged from 2.88% to 10.06%. The expression pattern of the HPRT gene followed a very similar pattern, although the variability between the groups was significantly lower: 1.9% in the group of two and 9.76% in the group of five.

The expression level of the HIF1α gene in the studied groups is lower compared to the individually cultured BMDMs group, with the lowest average expression occurring in the group of three. The coefficients of variation were not high and varied slightly between groups (3.18–7.77%). The expression of the Ly6C gene and the Glut1 gene seems to be similar for all experimental combinations. The expression levels of these genes are relatively stable. Regarding the Ly6C expression, the coefficients of variation do not differ much between the studied groups (5.04–9.23%). However, a higher variability is observed for Glut1 expression. The coefficients of variation range from 0.92% (group of two) and 3.6% (group of four) to 13.4% (individuals group) or 14.53% (group of three). The group with the highest average expression of CD11c is the group of five, while the individuals group showed the highest variability (21.98%). Other groups showed slightly elevated expression levels compared to the individuals group. Their coefficients of variation ranged from 4.71% (group of four) to 13.55% (group of two). The expression of the iNOS gene, which had the lowest expression among the examined genes, was relatively stable and did not significantly differ between experimental groups. Coefficients of variation were not high and ranged from 1.18% in the group of two to 7.33% in the group of individuals. The average expression of the CD86 gene was similar for each of the studied groups except for the group of five, which exhibited lower expression levels and also the highest variability (183.7%). Compared to the groups of two, three, and four, the individuals group was the most variable (18.29%). Other groups have coefficients of variation ranging from 1.69% to 3.36%. The ANOVA test showed a difference only for HPRT and HIF1α expression. 

### 2.4. Cytokine Secretion

**Figure 4:**
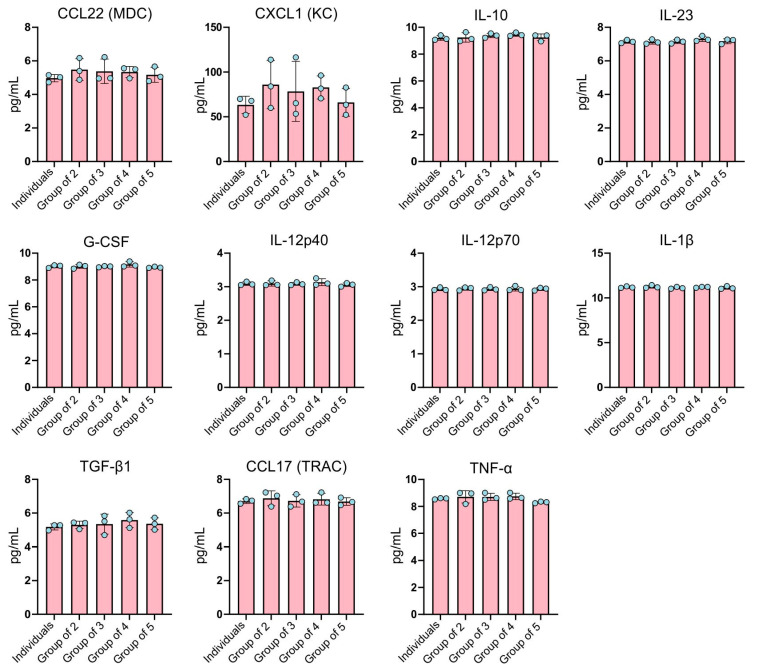
Cytokine secretion levels by BMDMs across different experimental conditions, presented as the mean ± SD (pg/mL). Data represent the average of three independent biological replicates. Individual data points are shown as dots. Statistical analysis was performed using one-way ANOVA with Tukey’s multiple comparisons test.

The cytokine secreted at the highest concentration was CXCL1 (KC) cytokine (Figure 4). 

All experimental groups exhibited either an equal or higher production of CXCL1 (KC) compared to single-cultured BMDMs. The group of three exhibited the highest variability (coefficient of variance at 42.86%) and the individuals group the lowest (15.23%). There were no statistical differences between the groups. The variability between groups as well as between biological replicates were relatively low for other cytokines, but the level of these cytokines secreted to the culture medium was also relatively low (Figure 4). The lowest concentrations were noted for IL-12p40 and IL-12p70, which is not surprising since these cytokines are produced after antigenic stimulation. The average concentrations of these cytokines were around 3 pg/mL and coefficients of variation were up to 3.2%. A slightly higher concentration was measured for TGF-β1 and CCL22 (MDC), around 5 pg/mL, with the highest coefficients of variation for the group of three for both cytokines, 11.09% and 13.61%, respectively. The concentrations of cytokines at the level of around 7 pg/mL were for CCL17 (TRAC) and for IL-23, and there were also no notable differences between groups. The highest coefficients of variation for CCL17 were for the group of two (6.39%) and the lowest were for the individuals group (2.08%); for IL-23, the highest coefficients of variation were for the group of four (2.16%) and the lowest were also for the individuals group (1.36%). However, in both cases the differences were not statistically significant. The secretion of TNF-α, G-CSF, and IL-10 was slightly higher and around 9 pg/mL in all experimental groups. The highest coefficients of variation were 5.29% (group of two), 2.19% (group of four), and 3.78% (group of two) for TNF-α, G-CSF, and IL-10, respectively. The lowest coefficients of variation were 0.56% (individuals), 0.51% (group of five), and 1.41% (group of four) for TNF-α, G-CSF, and IL-10, respectively. For IL-1β, concentrations above 10 pg/mL were observed, but these were also without differences between groups and with low variations (0.55–1.44%). 

### 2.5. Capacity for Phagocytosis and Nitric Oxide (NO) Levels in Culture Medium

**Figure 5:**
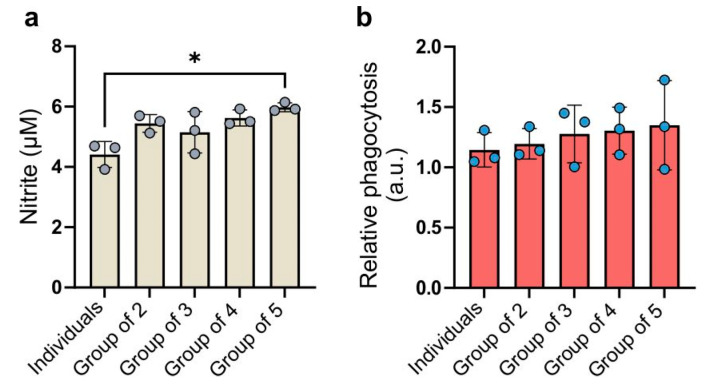
Functional characteristics of BMDMs. (**a**) Nitrite concentration in the culture medium (µM), presented as the mean ± SD across different experimental conditions; (**b**) Phagocytic capacity of BMDMs, expressed as relative values and presented as the mean ± SD for various experimental conditions. Data are presented from three biological replicates. Individual data points are shown as dots. Statistical analysis was performed using one-way ANOVA with Tukey’s multiple comparisons test; * indicates *p* < 0.05.

The levels of nitrite secretion varied among the experimental groups (Figure 5a). 

Each combined group exhibited a higher secretion of nitric oxide into the culture medium compared to individually cultured macrophages. The highest concentration of nitric oxide was detected in the culture medium from cells in the group of five. The ANOVA test showed a statistical significance between the group of five and group of individuals but the difference was negligible. The group of three exhibited the highest coefficient of variation (13.35%), while the group of five had the lowest (2.46%). Cells cultured separately exhibited the lowest phagocytic capacity (Figure 5b). In the groups of two, three, and four, the average phagocytic capacity gradually increased relative to the individuals group. In the group of five, in which BMDMs from five individuals were combined, the average phagocytosis intensity was the highest. The level of variability in phagocytosis intensity was substantial not only between biological replicates but also between combinations within each replicate. This may suggest that phagocytosis intensity is highly dependent on individual characteristics. The ANOVA test indicated that the differences between the studied groups were not statistically significant. The coefficients of variation in the groups ranged from 10.51% (group of two) to 27.44% (group of five).

### 2.6. Expression of Cell Surface Markers on Human Macrophages

**Figure 6:**
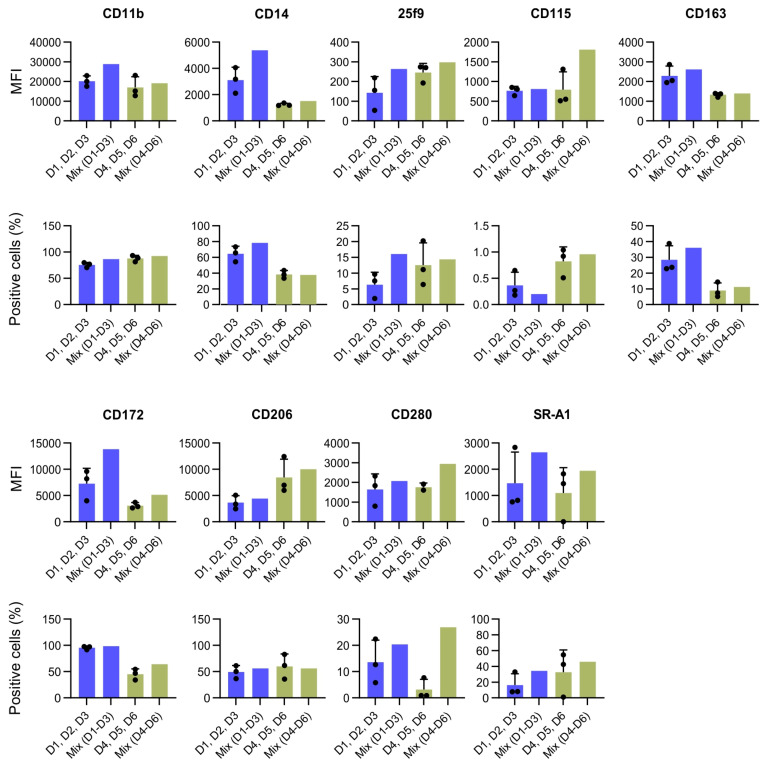
Expression of surface markers on hMDMs in experimental combinations. The bar graphs show the mean ± SD fluorescence intensity (MFI) and the percentage of positive cells for various surface markers across different groups and individuals. Each marker is represented by two graphs: the upper row for the MFI and the lower row for the percentage of positive cells. The markers analyzed include CD11B, CD14, 25f9, CD115, CD163, CD172, CD206, CD280, and SR-A1. Data are presented as the mean ± standard deviation (SD) from three biological replicates. Individual data points are shown as dots.

To further delineate the impact of pooling cells from multiple donors on macrophage phenotypes, we evaluated the expression of several surface markers on human monocyte-derived macrophages (hMDMs) cultured from individual donors compared to those cultured as pooled mixes (Figure 6). Two groups of donors were analyzed: in the first, cells from D1, D2, and D3 were compared to a mix of these three donors (Mix D1–D3; shown in blue bars), and in the second, cells from D4, D5, and D6 were compared to their pooled mixture (Mix D4–D6; shown in green bars). We assessed macrophage characteristics using both the mean fluorescence intensity (MFI) and the percentage of cells positive for each marker. For core macrophage lineage markers, such as CD11b, the pooled cultures (Mix D1–D3) exhibited higher MFI values (reaching approximately 20,000–28,000) compared to individual donors, which generally ranged from ~15,000 to 20,000. In the second set of donors (D4–D6), mixing also led to stable or enhanced CD11b expression, both in terms of the MFI and the proportion of positive cells. Similarly, CD172a (SIRPα), another key receptor associated with macrophage lineage, displayed noticeably increased MFI values in pooled cultures, suggesting that incorporating genetically diverse donor cells can drive a more-uniform and -elevated expression of core macrophage markers.

Pooling also influenced markers related to specialized macrophage functions. For example, SR-A1, a scavenger receptor, and CD280, potentially involved in innate immune signaling, both showed substantial increases in the MFI upon mixing. SR-A1, which was expressed at relatively low levels in single-donor cultures, rose to much higher intensities in the mixes. Similarly, CD280 reached MFI values surpassing those found in individual donors. These observations imply that coculturing macrophages from multiple donors can enhance the overall functional readiness or diversity of these populations. Not all markers responded to mixing with a pronounced increase. For example, CD163 and CD206, which can reflect alternative activation states, exhibited more-variable or -modest changes upon coculturing. In some cases, the pooled cultures yielded intermediate values, suggesting that donor-to-donor differences were “averaged out” rather than synergistically enhanced. Examining the percentage of cells expressing each marker further supported the notion that combining donors can produce a more-uniform or -enhanced phenotype. For instance, markers that were less prevalent in individual cultures, such as CD280 or SR-A1, became substantially more common in the pooled mixes, indicating that coexisting macrophage subsets may collectively shift the population toward a more-activated state. In contrast, some markers, including CD14 and CD163, displayed minimal changes in their positive cell percentages, suggesting that certain phenotypic traits remain relatively stable even when diverse donor populations are combined.

Overall, the data presented in Figure 6 demonstrate that coculturing hMDMs from multiple donors can lead to either an elevated baseline expression of key lineage and functional markers or the “averaging” of intermediate phenotypes. These shifts underscore the potential of mixing genetically and environmentally distinct donor cells to achieve more-robust, more-reproducible, and potentially more-functionally versatile macrophage populations.

## 3. Discussion

The challenges associated with low accuracy and reproducibility in preclinical studies often stem from significant inter-individual variability within immune cell populations [24,25,26,27]. Macrophages, distributed throughout the body as first responders to homeostatic perturbations, are no exception. A deeper understanding of their phenotypic and functional heterogeneity is essential for improving the translational relevance of in vitro models [28,29]. Various studies conducted on standard mouse models have been characterized by low accuracy and reproducibility of results, leading to unsatisfactory levels of success in clinical research [24,25,26,27]. One possible cause of this problem is the significant inter-individual variability of immune cell populations [28]. Numerous findings have demonstrated that such variability significantly impacts various pathophysiological processes [28,29]. Although there are limited references on the natural diversity of macrophage polarization in health and disease, it has been shown that accounting for immunological diversity within a heterogeneous population can increase result reproducibility [29].

In this study, we examined mouse bone marrow-derived macrophages (BMDMs) and human monocyte-derived macrophages (hMDMs) to determine how pooling cells from multiple donors influences macrophage phenotypes. We hypothesized that combining cells from different individuals would reduce inter-individual variability and thereby enhance the reproducibility of experimental outcomes. To test this, we evaluated the effects of donor heterogeneity on macrophage polarization, cytokine expression, and nitric oxide production: key indicators of immune function. Although we used a single inbred mouse strain (BALB/c) for the murine experiments—minimizing genetic variability—it is important to note that even inbred strains can display phenotypic differences driven by epigenetic changes, stochastic immune maturation, microbiome diversity, and other subtle environmental factors. Such factors may lead to striking phenotypic divergence—even under seemingly identical conditions—which has been well-documented, yet their direct impact on macrophage phenotypes remains understudied [30,31,32]. Nevertheless, studying macrophages from individuals with diverse genetic backgrounds is also crucial, especially in the era of cell therapies, where there is a growing need for allogeneic treatments to improve efficiency, accessibility, and cost-effectiveness without compromising safety. Although similar studies have started to appear in the literature [30,31,32], most have focused on stem cells rather than on macrophages. 

In the mouse model, BMDMs isolated from individual donors or pooled from two, three, four, or five donors did not exhibit significant differences in viability, size, or surface marker expression, remaining consistent with results reported by other researchers [15,33,34,35]. The consistency in core macrophage markers such as CD11b and F4/80, as well as stable cell yields, underscores that pooling does not disrupt fundamental macrophage identity. Furthermore, our study did not detect any significant shifts in macrophage activation status based on the expression levels of M1 or M2 markers, or other key receptors such as TLR4 and CD64, indicating that donor variability and pooling strategies do not significantly impact macrophage phenotype or gene expression profiles [36,37]. Similarly, functional outputs, including cytokine secretion, were in line with values reported in the literature [38,39,40]. BMDMs in our experiments secreted typical amounts of nitric oxide [39], and NO production showed no significant changes apart from a non-significant trend toward increased levels in larger pooled groups. These results indicate that while pooling cells increases overall sample size and can help reduce donor-to-donor variability, it does not compromise the baseline phenotype or functionality of murine BMDMs.

Our findings with pooled hMDMs underscore the potential advantages of combining cells from different donors to mitigate the pronounced variability that often characterizes human samples. Unlike murine donors from standardized inbred strains, human donors can vary extensively in genetics, immune history, and environmental exposures, which can lead to large standard deviations for certain surface markers and make it challenging to rely on a single donor for highly specialized or reproducible macrophage phenotypes. Indeed, we observed differences when comparing donor group 1 (D1–D3) to donor group 2 (D4–D6), reflecting the inherent variability in human populations. By pooling monocytes, we found that the expression of core markers, such as CD11b and CD172a, often became more uniform or elevated, suggesting a stabilizing effect on key macrophage traits. Functional markers such as SR-A1 and CD280 also showed significant increases in pooled cultures, indicating a heightened or more-cohesive activation state. However, markers like CD163 and CD206 displayed only moderate or variable changes, highlighting that not all phenotypes respond equally to coculturing. From a practical perspective, this averaging effect could be highly beneficial for applications requiring consistent, standardized macrophage products: for example, in large-scale cell therapy manufacturing or translational research, where uniformity and reproducibility are critical. 

Taken together, results from both murine and human systems demonstrate that pooling macrophages from different donors can help mitigate inter-individual variability, potentially improving reproducibility and experimental consistency. This strategy may be particularly valuable for high-throughput screens, drug testing, and comparative studies where consistency and translational applicability are paramount. At the same time, the absence of significant phenotypic shifts suggests that single-donor cultures remain important for research focusing on subtle donor-specific differences or personalized immunological signatures.

In conclusion, our combined approach—assessing both murine BMDMs and human hMDMs—demonstrates that pooling cells from multiple donors reduces variability without fundamentally altering macrophage identity, activation state, or functional capacity. These insights support the feasibility of using pooled macrophage cultures to enhance reproducibility in immunological research, guiding future efforts to standardize protocols and refine experimental models, and ultimately contributing to more-reliable and -translatable outcomes in both basic and clinical research contexts.

## 4. Materials and Methods

### 4.1. Mouse Bone Marrow-Derived Macrophages

The BALB/c mice used in this study were born on 18 August 2022, 15 November 2022, 24 January 2023, 1 March 2023, and 4 April 2023 and were obtained from the Mossakowski Medical Research Institute, Polish Academy of Sciences in Warsaw (Poland). The mice were put into the following groups: A, B, C, D, and E (cells from each mouse were grown in separate plates; after the analysis the mean results were calculated and labeled as “Individuals”), the group of two (mouse A and B), the group of three (mouse A, B, and C), the group of four (mouse A, B, C, and D), and the group of five (mouse A, B, C, D, and E). For each biological replicate, 5 mice were sacrificed, making a total of 15 mice. 

L929 cells were grown to full confluence in 150 cm² flasks (Corning, New York, NY, USA) using DMEM/F12 (Gibco, San Jose, CA, USA) medium enriched with 10% heat-inactivated FBS (Cytiva/HyClone, Marlborough, MA, USA) and 100 U/mL penicillin/streptomycin (Gibco, USA). After a period of 7 days, the conditioned medium from the L929 cells was harvested, filtered, and subsequently stored at −80 °C until needed.

The bone marrow cells were harvested from murine femurs and tibias and were seeded onto 90 mm-diameter non-treated polystyrene Petri dishes (Bionovo, Legnica, Poland) at a density of 2 × 10^6^ cells per plate in 5 mL of culture medium. The medium used was DMEM/F12 (Gibco, USA) supplemented with 10% FBS (Cytiva/HyClone, USA), antibiotics (100 U/mL penicillin/streptomycin, Gibco, USA), and 20% of L929-conditioned medium as the source of M-CSF. Three days after seeding, an additional 5 mL of culture medium was added. After two days, the medium was replaced with 10 mL of fresh culture medium and the cells were cultured for another 2 days.

### 4.2. Human Monocyte-Derived Macrophages (hMDMs)

hMDMs were generated from buffy coats procured from the Regional Blood and Hemotherapy Centre in Warsaw (Poland), with the samples purchased for research purposes. These buffy coats were supplied anonymously, adhering to Polish legal regulations (§13 Dz.U.1997.106.681), ensuring that they could not be linked to any specific donor. Polish law aligns with Directive 2002/98/EC of the European Parliament and the Council, dated 27 January 2003, which defines quality and safety standards for the collection, testing, processing, storage, and distribution of human blood and its components. In accordance with these guidelines, the Committee on the Ethics of Research Involving Human Subjects at the Warsaw University of Life Sciences was informed about their use.

Human peripheral blood mononuclear cells (PBMCs) were isolated through density gradient centrifugation (30 min, 21 °C, 400× *g*, no acceleration or braking) using Histopaque-1077 (Sigma-Aldrich, St. Louis, MO, USA). Monocytes were then obtained using the Pan Monocyte Isolation Kit (Miltenyi Biotec, Los Angeles, CA, USA), placed into sterile 90 mm Petri dishes (Promed, FL Medical, Padova, Italy) at a density of 6 × 10^6^ cells per plate, and cultured for five days in TexMACS medium (Miltenyi Biotec, USA) supplemented with 100 ng/mL M-CSF (Miltenyi Biotec, USA).

### 4.3. Real-Time PCR

Total RNA from one million cells suspended in Trizol (LifeTechnologies, Waltham, MA, USA) was isolated on silica columns (A&A Biotechnology, Gdańsk, Poland) followed by the cDNA synthesis (Roche, Basel, Switzerland). A quantitative RT-PCR was performed using a Universal SYBR^®^ Green Super Mix (Biorad, Hercules, CA, USA) and a Dx Real-Time PCR System with the use of software Aligent Aria (Agilent, v. 1.71, Santa Clara, CA, USA). Data were analyzed using the comparative Ct method. Sequences of key genes involved in monocyte/macrophage biology—B2M (reference gene), HIF1a, Sod1, Glut1, Itgb2, Ly6C, Hprt, iNOS, CD36, CD14, CD206, and CD11b—were obtained from Primer Bank (Table 1). Results were normalized to the B2M housekeeping gene. This analysis has been performed on the cells obtained from two various cell sources in triplicate.

### 4.4. Measurement of Surface Marker Expression Levels

Cells were counted to a concentration of 1 × 10^6^ cells and transferred into flow cytometry tubes. A total of 400 µL of phosphate-buffered saline (Gibco, USA) was used to suspend the cells. To identify dead cells, 1 µL of Zombie Aqua dye (BioLegend, San Diego, CA, USA) was added to the cell suspension. The tubes were incubated for 10 minutes in the dark. After incubation, 1 mL of PBS (Gibco, USA) was added to each tube, followed by centrifugation at 450× *g* for 5 minutes. The supernatant was discarded, and the cell pellet was resuspended in 35 µL of 5% rat serum (Sigma-Aldrich, USA) or FcR blocker (Miltenyi Biotec, USA) for hMDMs to block non-specific binding sites; this was incubated for 10 min at 4 °C. Subsequently, each antibody was added separately to the tubes at a 1:100 dilution. The cells were incubated with the antibodies for 30 min at 4 °C. After antibody binding, cells were washed by adding 1 mL of PBS (Gibco, USA), followed by centrifugation at 450× *g* for 5 min. The supernatant was aspirated, and cells were resuspended in 100 µL of PBS (Gibco, USA). The labeled cells were analyzed using a Canto II flow cytometer (BD Biosciences, San Jose, CA, USA) operated with FACSDiva™ software Version 8.0.2 (BD Biosciences, USA). A minimum of 30,000 cells per sample were analyzed. Data were analyzed using FlowJo version 10.1 (FlowJo, LLC Data Analysis Software, Bend, OR, USA). The gating strategy is presented in Supplementary Figure S1. Bone marrow-derived macrophages (BMDM) in the samples were identified using the following antibodies, each targeting specific surface markers: Anti-CD11b-FITC (Invitrogen, San Diego, CA, USA); Anti-TLR2-Pacific Blue (BD, Franklin Lakes, NJ, USA); Anti-TLR4-APC (Invitrogen, USA); Anti-F4/80-APC-Cy7 (BioLegend, USA); Anti-CD64-PE-Cy7 (BioLegend, USA); and Anti-Ly6C-APC (Invitrogen, USA). Human monocyte-derived macrophages (hMDM) in the samples were identified using the following antibodies, each targeting specific surface markers: Anti-CD11B-FITC (Invitrogen, USA), Anti-CD14-PerCP5.5 (BD, USA), Anti-CD163-PE (BD, USA), Anti-CD206-BV421 (BD, USA), Anti-25f9-APC (eBioscience, San Diego, CA, USA), Anti-CD115-FITC (Biolegend, USA), Anti-CD280-PE (BD, USA), Anti-SR-A1-BV421 (BD, USA), and Anti-CD172-APC (Biolegend, USA).

### 4.5. Phagocytosis Assay

Phagocytosis assays were performed with the VybrantTM Phagocitosis Assay Kit (Molecular Probes, Eugene, OR, USA) according to manufacturer’s protocol. Cells were plated on 96-well plates for 1 h. The negative control was the DMEM medium (Gibco, USA). Cells were treated with E.coli solution prepared by sonication with HBSS buffer (Gibco, USA). After incubation, the medium from above the cells was collected and 100 µL of Trypan blue solution (LifeTechnologies, USA) was added to each well, incubated for 1 min and aspirated. The values were read using the Tecan Infinite 200 Pro Microplate Reader (Männedorf, Switzerland) and the Tecan i-control software (v. 2.0.10.0). The excitation wavelength was set to 480 nm, the emission wavelength was set to 520 nm, the gain was set to Optimal, and the Z-Position was calculated from well A1. This analysis has been performed in five repetitions.

### 4.6. Evaluation of Cytokine Secretion

Assessment of cytokine secretion was performed using the LEGENDplexTM Multi-Analyte Flow Assay Kit (BioLegend, USA). The following cytokines were tested: KC, TGF-β1, IL-18, IL-23, MDC, IL-10, IL-12p70, IL-6, TNF-α, G-CSF, TARC, IL-12p40, and IL-1β.

First, the liquid above the cell culture was gathered and spun in a centrifuge for 10 min at 5000× *g*, at 4 °C. This liquid was then transferred into small tubes, each holding 1.5 mL. For each sample, the liquid was diluted by mixing 50 µL of it with 50 µL of Assay Buffer. Additionally, seven standard concentration solutions were prepared, ranging from 10,000 pg/mL down to 2.44 pg/mL, with each concentration being four-times lower than the previous one. Next, onto a plate, 25 µL of Assay Buffer, 25 µL of the prepared liquid from earlier, and 25 µL of mixed beads were added to each well. The plate was then gently shaken for 2 h at RT and 800 rpm. Following this, the plate underwent another spin in the centrifuge, this time for 5 min at 250× *g*, after which the excess liquid was removed. The wells were then washed with 200 µL of Wash Buffer, spun again, and the excess liquid removed. To each well, 25 µL of detection antibodies was added, and the plate was shaken for 1 hour at room temperature and 800 rpm. Afterward, 25 µL of SA-PE was added to each well and the plate was shaken for 30 min at RT and 800 rpm. Another round of centrifugation followed, lasting 5 min at 250× *g*, and the excess liquid was removed. The wells were washed again with 200 µL of Wash Buffer, spun, and excess liquid removed. Finally, 150 µL of Wash Buffer was added to each well, and the solutions were transferred to cytometry tubes. The results were then analyzed using a Canto II flow cytometer (BD, USA) operated by the FACSDiva™ program. Each sample had 3000 beads analyzed, with the retraction speed set to low.

Dose–response curves for each cytokine were modeled using the four-parameter logistic (4PL) function. The 4PL model was fitted using the “drc” package in R (R Core Team, v. 4.3, Vienna, Austria). Each cytokine‘s response data were fitted independently to a 4PL model and model fits were visually inspected using diagnostic plots to ensure appropriate data fitting.

### 4.7. NO Production Assay

The Total Nitric Oxide Assay Kit (Invitrogen, USA) was used for the measurement. On the seventh day of culture, the supernatant culture medium was collected and diluted 1:2 with 1× Reagent Diluent. Appropriate reagents for the reaction and a standard curve were prepared. Absorbance measurements were conducted at a wavelength of 550 nm. The concentration of total nitric oxide was determined by first subtracting the average absorbance of the blank from the obtained absorbances. Subsequently, the average absorbance of the zero sample was subtracted from all averaged test and reference samples. A standard curve was then generated based on the absorbance values of standard samples. Finally, the total concentration of nitric oxide was calculated by substituting the absorbance values into the equation of the standard curve.

### 4.8. Statistical Analyses

The statistical analysis of the results was carried out using the GraphPad Prism program (v. 9.5.1, USA). To assess the relationship between more than two study groups, ANOVA and coefficient of variation analyses were conducted. A significance level (p) of less than or equal to 0.05 was considered statistically significant. For interpretation, the following conventions were used: **** denotes *p* < 0.0001, *** indicates *p* < 0.001, ** signifies *p* < 0.01, and * represents *p* < 0.05. Given the biological context and practical limitations of our study, including ethical considerations, cost, time constraints, and the homogeneity of inbred mice, we conducted the experiment using three independent biological replicates. This aligns with minimal recommendations for group sizes in similar studies, as outlined by Wan Nor Arifin and Wan Mohd Zahiruddin (2017), who suggest the formula for minimum sample size *n* = 10/*k* + 1, where *k* is the number of groups [41]. For our study consisting of five groups, this formula yields a minimum of three replicates.

## Figures and Tables

**Table 1:** Sequence of starters used for the RT-PCR analysis.

Gene	Sequence 5’	Sequence 3’
B2m	5’-TTCTGGTGCTTGTCTCACTGA-3’	5’-CAGTATGTTCGGCTTCCCATTC-3’
Hif1a	5’-CCACAGGACAGTACAGGATG-3’	5’-TCAAGTCGTGCTGAATAATACC-3’
Sod1	5’-TTGTTTCTCATGGACCAC-3’	5’-GATCGTGTGATCTCACTCTC-3’
Glut1	5’-CAGTTCGGCTATAACACTGGTG-3’	5’-GCCCCCGACAGAGAAGATG-3’
Itgb2	5’-CTTTCCGAGAGCAACATCCAGC-3’	5’-GTTGCTGGAGTCGTCAGACAGT-3’
Ly6C	5’-GCAGTGCTACGAGTGCTATGG-3’	5’-ACTGACGGGTCTTTAGTTTCCTT-3’
Hprt	5’-TCAGTCAACGGGGGACATAAA-3’	5’-GGGGCTGTACTGCTTAACCAG-3’
iNOS	5’-GTTCTCAGCCCAACAATACAAGA-3’	5’-GTGGACGGGTCGATGTCAC-3’
CD36	5’-GTCTTCCCAATAAGCATGTCTCC-3’	5’-ATGGGCTGTGATCGGAACTG-3’
CD86	5’-TGTTTCCGTGGAGACGCAAG-3’	5’-TTGAGCCTTTGTAAATGGGCA-3’
CD14	5’-CTCTGTCCTTAAAGCGGCTTAC-3’	5’-GTTGCGGAGGTTCAAGATGTT-3’
CD206	5’-CGGAATTTCTGGGATTCAGCTTC-3’	5’-CGGAATTTCTGGGATTCAGCTTC-3’
CD11b	5’-ATGGACGCTGATGGCAATACC-3’	5’-TCCCCATTCACGTCTCCCA-3’
CD11c	5’-GCACACTGTGTCCGAACTCA-3’	5’-CTGGATAGCCTTTCTTCTGCTG-3’

## Data Availability

The raw data from this study are available upon request from the corresponding author.

## Supplementary Materials

The following supporting information can be downloaded at: https://www.mdpi.com/article/10.3390/ijms26031272/s1.
