# Supplementary material for: Unveiling the Phenotypic Variability of Macrophages: Insights from Donor Diversity and Pooling Strategies
Source: Int J Mol Sci. 2025 Jan 31;26(3):1272. doi: 10.3390/ijms26031272 (PMC11818227; doi:10.3390/ijms26031272)
Supplement: Supplementary file 1 [file ijms-26-01272-s001.zip › ijms-3410467-Supplementary.pdf]

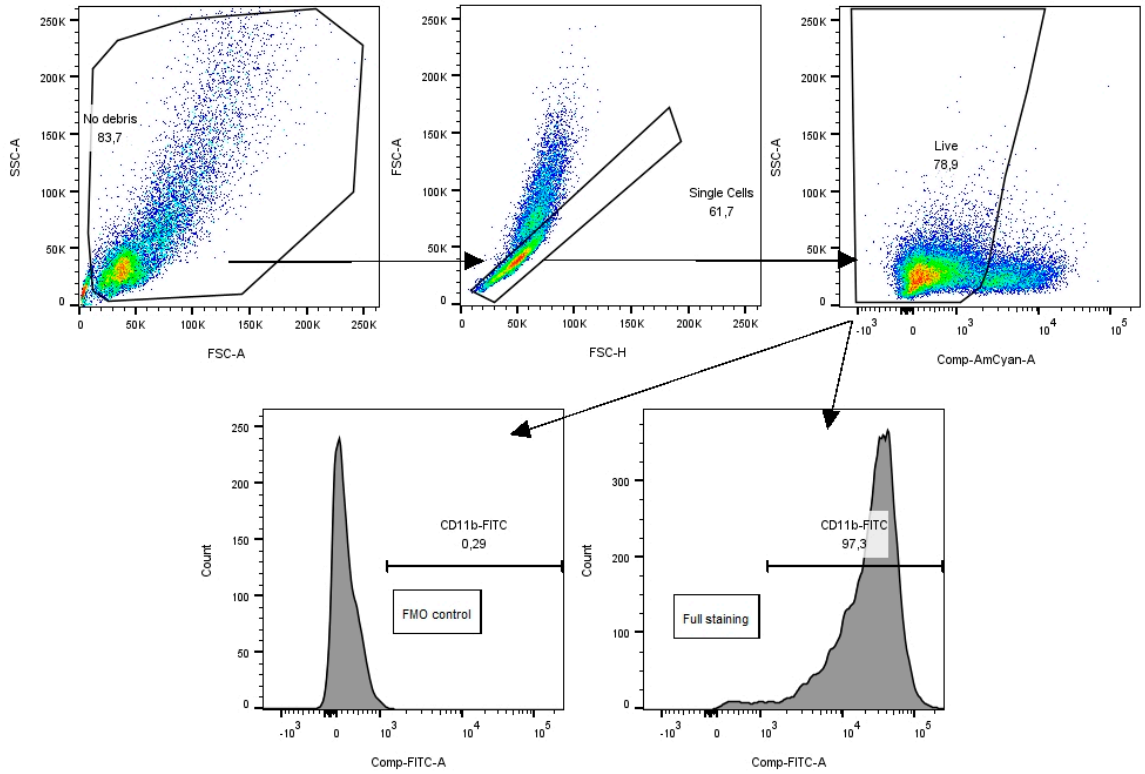

Figure S1. Gating Strategy. Representative gating strategy for the analysis of macrophage markers by flow cytometry. Events were first gated to exclude cell debris, followed by gating for singlets. Next, dead cells were excluded, and the mean fluorescence intensity (MFI) of each marker was measured within the live cell population. Specific marker-positive cells were identified using fluorescence-minus-one (FMO) controls.
